# Does Dialysis Type Matter? Re-Evaluating Prognosis in UTUC Patients Following Surgery

**DOI:** 10.3390/cancers18162670

**Published:** 2026-08-18

**Authors:** Yi-Ying Hsieh, I-Hsuan Alan Chen, Chia-Cheng Yu, Chao-Hsiang Chang, Chin-Chung Yeh, Wei-Ming Li, Hung-Lung Ke, Bor-En Jong, Yi-Ju Chou, Chung-You Tsai, Pai-Yu Cheng, Marcelo Chen, Wun-Rong Lin, Vincent F. S. Tsai, Yao-Chou Tsai

**Affiliations:** 1 Department of Urology, National Taiwan University Hospital, Taipei 100225, Taiwan; connie830818@gmail.com; 2 Division of Urology, Department of Surgery, Taipei Tzu Chi Hospital, The Buddhist Tzu Chi Medical Foundation, New Taipei City 231057, Taiwan; 3 Division of Urology, Department of Surgery, Kaohsiung Veterans General Hospital, Kaohsiung 813414, Taiwan; 4 Department of Urology, China Medical University and Hospital, Taichung 404327, Taiwan; 5 School of Medicine, China Medical University, Taichung 404333, Taiwan; 6 Department of Urology, Kaohsiung Medical University Gangshan Hospital, Kaohsiung 820004, Taiwan; 7 Department of Urology, Kaohsiung Medical University Hospital, Kaohsiung 807378, Taiwan; 8 Department of Urology, School of Medicine, College of Medicine, Kaohsiung Medical University, Kaohsiung 807378, Taiwan; 9 Department of Urology, Ministry of Health and Welfare Pingtung Hospital, Pingtung 900075, Taiwan; 10 Graduate Institute of Medicine, College of Medicine, Kaohsiung Medical University, Kaohsiung 807378, Taiwan; 11 Divisions of Urology, Department of Surgery, Far Eastern Memorial Hospital, New Taipei City 22060, Taiwan; 12 Department of Electrical Engineering, Yuan Ze University, Taoyuan 320808, Taiwan; 13 Department of Urology, MacKay Memorial Hospital, Taipei 104217, Taiwan; 14 Department of Medicine, Mackay Medical College, New Taipei City 252307, Taiwan; 15 Department of Nursing and Management, Mackay Junior College of Medicine, Nursing, and Management, Taipei 112105, Taiwan; 16 School of Medicine, Buddhist Tzu Chi University, Hualien 970374, Taiwan; 17 Department of Urology, Taipei Medical University Hospital, Taipei Medical University, Taipei 110301, Taiwan

**Keywords:** upper tract urothelial carcinoma, perioneal dialysis, hemodialysis, radical nephroureterectomy, propensity score, survival

## Abstract

Background: Patients with end-stage renal disease (ESRD) undergoing radical nephroureterectomy (RNU) for upper tract urothelial carcinoma (UTUC) represent a uniquely high-risk population. While prior studies have demonstrated worse postoperative outcomes among dialysis-dependent patients, no study has systematically compared oncological outcomes between peritoneal dialysis (PD) and hemodialysis (HD) modalities following RNU. This multicenter study evaluates whether dialysis modality (peritoneal dialysis versus hemodialysis) acts as an independent prognostic factor following radical nephroureterectomy. Methods: Using the Taiwan UTUC Collaboration Group Registry—a multicenter, nationwide database comprising 21 tertiary and regional medical centers —we identified 350 ESRD patients who underwent RNU for UTUC between September 1988 and December 2023. Patients were categorized by dialysis modality at the time of surgery: 310 on HD and 40 on PD. Propensity score overlap weighting was applied to account for baseline differences. Multivariate Cox proportional hazards and Fine-Gray competing risk regression models were utilized to evaluate overall survival (OS), cancer-specific survival (CSS), progression-free survival (PFS), and non-UTUC mortality. Results: After overlap weighting, PD was independently associated with significantly worse OS (HR = 2.34, 95% CI 1.29–4.25, *p* = 0.005) and PFS (HR = 2.06, 95% CI 1.16–3.66, *p* = 0.013) compared to HD and exhibited a trend toward worse CSS in univariate analysis (log-rank *p* = 0.094). Survival curve divergence between PD and HD was most pronounced from 12 to 24 months post-surgery onward. Notably, this survival disadvantage persisted despite PD patients being significantly younger (mean age 58.8 vs. 65.1 years) and receiving adjuvant chemotherapy more frequently (20.0% vs. 6.5%). PD was also independently associated with higher non-UTUC mortality on multivariate competing risk analysis (sHR = 2.17, 95% CI 1.22–3.88, *p* = 0.009). Conclusions: In this first multicenter systematic comparison of PD versus HD patients undergoing RNU for UTUC, PD modality was independently associated with worse OS and PFS and exhibited a trend toward worse CSS in univariate analysis, compared to HD. This survival disadvantage persists despite favorable baseline characteristics and higher rates of adjuvant chemotherapy. We hypothesize these outcomes may be driven by a dual vulnerability: impaired systemic tumor control and elevated non-cancer mortality following surgical disruption of the peritoneal environment. To mitigate these risks, prioritizing minimally invasive or retroperitoneal surgical approaches to preserve peritoneal integrity, combined with modality-specific multidisciplinary surveillance, is recommended.

## Simple Summary

Study Objective: Does the type of dialysis a kidney failure patient uses affect their chances of surviving cancer surgery? Patients with severe kidney failure who need surgery for upper tract urothelial carcinoma already face very high health risks. However, which type of dialysis may be more suitable for these patients remains unclear. We aimed to find out whether peritoneal dialysis (PD) or hemodialysis (HD) makes a real difference to how long these patients live and how well they recover. Study Results: The answer was clear. After carefully balancing background differences between the two groups, significantly worse overall survival (OS) and progression-free survival (PFS) were observed in patients on PD compared to those on HD. For cancer-specific survival (CSS), while the difference narrowly missed strict statistical significance, it demonstrated a strong and clinically meaningful trend toward worse outcomes in the PD cohort. Proposed Hypotheses: So why does PD seem to carry worse odds? We hypothesize that this survival disadvantage in PD patients is driven by a “dual vulnerability”. PD may quietly reshape the immune system, making it harder for the body to fight off any remaining cancer cells. At the same time, major abdominal surgery disrupts the exact tissue PD depends on, so dialysis often becomes less effective afterward. Once dialysis quality drops, patients can decline quickly, becoming more vulnerable to non-cancer causes of death, such as heart complications. Conclusions: Dialysis type is not a minor detail. It is a factor that can shape a cancer patient’s odds of survival. Clinicians must exercise heightened caution when managing PD patients, prioritizing minimally invasive or retroperitoneal surgical approaches to preserve peritoneal integrity whenever feasible.

## 1. Introduction

Upper tract urothelial carcinoma (UTUC) accounts for approximately 5–10% of all urothelial carcinomas. Taiwan has one of the highest incidences of UTUC worldwide, largely due to widespread exposure to aristolochic acid (AA) [1,2]. Recent genomic studies have further identified specific AA-related mutational signatures in Taiwanese patients [3,4]. Patients with end-stage renal disease (ESRD) are at increased risk of UTUC, and prior studies have shown that a history of dialysis is associated with higher recurrence rates, worse survival, and an increased risk of intravesical recurrence after surgery [5,6]. However, most previous studies have treated dialysis patients as a single group, without distinguishing between peritoneal dialysis (PD) and hemodialysis (HD) [6,7,8].

Contemporary risk-stratification frameworks have also evolved substantially in recent years: the 2025 EAU guidelines underwent a comprehensive revision of their risk-stratification chapter [9], and the AUA/Society of Urologic Oncology guidelines now stratify patients into low- and high-risk categories—further subdivided by favorable- and unfavorable-risk features—based on endoscopic, cytologic, pathologic, and radiographic findings [10]. However, these updated frameworks continue to offer limited guidance for the growing population of UTUC patients with concurrent ESRD, whose baseline surgical and oncological risk profile is already substantially altered by dialysis-related physiological changes.

PD and HD have different impacts on the peritoneal environment, immune function, systemic inflammation, and cardiovascular stress, which may lead to different oncological outcomes after radical nephroureterectomy (RNU) [11,12]. Given these modality-specific physiological differences, it is plausible that PD and HD patients may experience distinct postoperative trajectories following RNU. To date, studies comparing postoperative outcomes between PD and HD patients are scarce. Most existing literature has compared dialysis patients as a single group against non-dialysis controls, without distinguishing between PD and HD modalities [13]. In addition, no study has systematically compared oncological outcomes between PD and HD patients following RNU for UTUC. This represents a critical knowledge gap, particularly in regions such as Taiwan where both UTUC incidence and dialysis prevalence are among the highest worldwide.

To address this unmet need, the Taiwan UTUC Collaboration Group established a multicenter, nationwide registry enabling robust, modality-specific outcome analysis. Using this large dataset, our study aims to evaluate whether dialysis modality independently influences survival outcomes after RNU, and to clarify the clinical implications for perioperative management and postoperative surveillance in this high-risk population.

## 2. Methods

### 2.1. Study Design and Setting

This study was a multicenter retrospective cohort analysis conducted under the Institutional Review Board of Taipei Tzu Chi Hospital (no. 06-X34-105), with informed consent waived given the use of deidentified registry data and in strict compliance with local regulations. Patient data were obtained from the Taiwan UTUC Collaboration Group Registry, a large-scale multicenter database compiled from 21 tertiary and regional medical centers throughout Taiwan. The registry captures patients who underwent surgical treatment from 1 September 1988 through 31 December 2023; vital status and recurrence outcomes were updated through 31 December 2024. Statistical analyses were conducted between February and April 2026.

### 2.2. Inclusion and Exclusion Criteria 

**Figure 1:**
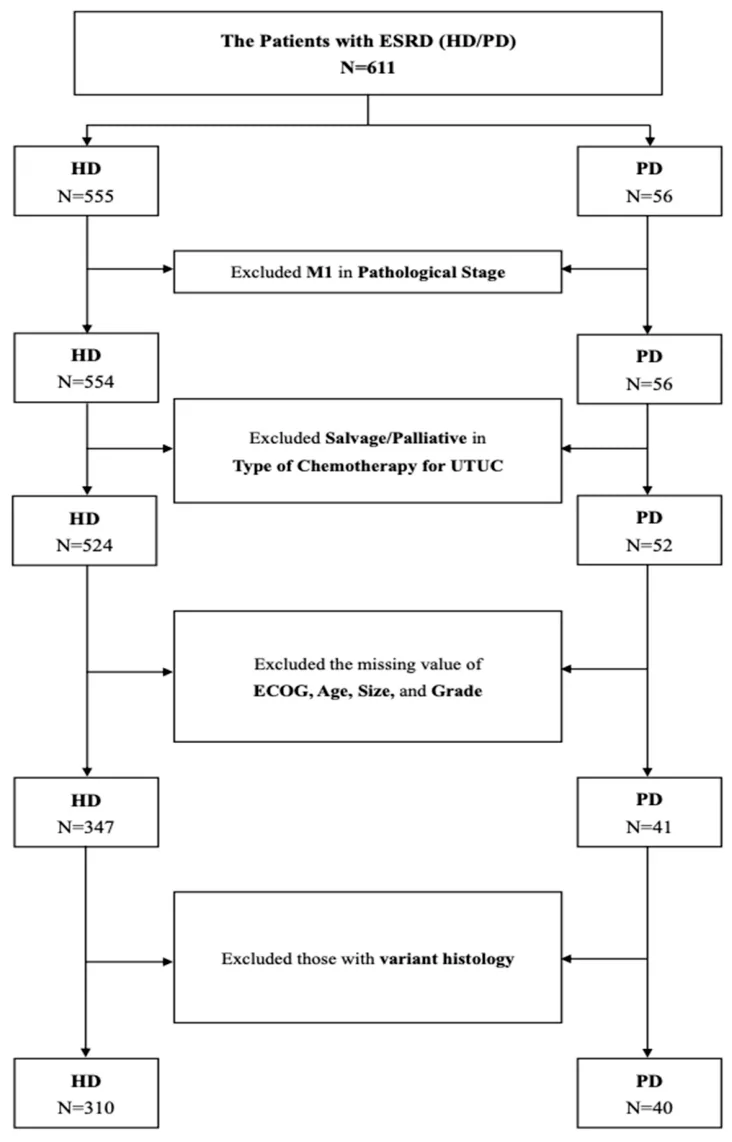
STROBE-compliant flow diagram of the study cohort. A total of 611 eligible patients with end-stage renal disease (ESRD) undergoing radical nephroureterectomy (RNU) for upper tract urothelial carcinoma (UTUC) who met the baseline clinical criteria (e.g., no documented switch in dialysis modality) were initially identified from the registry (555 on hemodialysis [HD] and 56 on peritoneal dialysis [PD]). Patients were sequentially excluded if they had distant metastasis (M1) at initial diagnosis, received salvage or palliative chemotherapy, had missing baseline clinicopathological data (including ECOG performance status, age, tumor size, or tumor grade) or had variant histology. The final analytic cohort comprised 350 patients (310 in the HD group and 40 in the PD group).

Eligible patients were adults aged 18 years or older with pathologically confirmed upper tract urothelial carcinoma who had undergone RNU at a participating center. To be included, patients must have been on dialysis for a minimum of 3 months before surgery. Patients were excluded for any of the following reasons: distant metastasis present at initial diagnosis, dialysis initiated only after surgery, coexisting active advanced malignancy, a documented switch in dialysis modality during the study period, administration of salvage or palliative chemotherapy, undergoing kidney-sparing procedures instead of standard RNU, those pathology was non-pure urothelial carcinoma (variant histology) or having missing baseline clinicopathological data (including ECOG performance status, age, tumor size, or tumor grade). A STROBE-compliant flow diagram detailing the patient selection process is provided in Figure 1. The index date was the date of RNU; follow-up continued until the occurrence of the event of interest, last clinical contact, or database closure, whichever came first.

### 2.3. Exposure, Outcomes, Covariates, and Data Sources

The primary exposure was dialysis modality at the time of RNU, categorized as peritoneal dialysis (PD) or hemodialysis (HD). The primary endpoint was cancer-specific survival (CSS), measured from RNU to death from UTUC. To ensure diagnostic accuracy, causes of death were strictly adjudicated by cross-linking with the Taiwan Ministry of Health and Welfare (MOHW) National Cause of Death Registry to unambiguously differentiate cancer-specific from non-cancer mortality.

Secondary endpoints comprised overall survival (OS) and progression-free survival (PFS), which were defined as follows:

- Overall Survival (OS): The time from the date of RNU to death from any cause.

Non-UTUC Mortality: Death from any cause other than UTUC (such as cardiovascular disease or infection).

- Progression-free survival (PFS): The conventional oncological outcome defined as the time from RNU to the first occurrence of disease progression—including locoregional recurrence (bladder cuff/lower ureter), regional lymph node metastasis, or distant metastasis, as determined by imaging (CT/MRI) or pathologic confirmation during surveillance—or death from any cause, whichever occurred first.
- Time to Progression (TTP): An alternative oncological outcome defined as the time from RNU to the first occurrence of disease progression—including locoregional recurrence (bladder cuff/lower ureter), regional lymph node metastasis, or distant metastasis, as determined by imaging (CT/MRI) or pathologic confirmation during surveillance. To evaluate the true oncological efficacy of surgery and exclude the confounding effect of non-cancer deaths, death before tumor progression was treated as a competing risk rather than a progression event.

For all outcomes, asymptomatic intravesical recurrences (excluding lower ureter/bladder cuff recurrences) and contralateral upper tract recurrences were managed endoscopically and not counted as systemic progression events.

Covariates collected at baseline included patient-level factors (age, sex, ECOG performance status, hypertension, diabetes mellitus, smoking history, preoperative creatinine level, previous nephroureterectomy and prior malignancy) and tumor-related characteristics (anatomical location, tumor size, multifocality, pathological T and N stage, histological grade, lymphovascular invasion, concomitant carcinoma in situ, preoperative hydronephrosis, preoperative urine cytology, and tumor necrosis). Perioperative data included receipt of chemotherapy, postoperative intravesical instillation, and the specific surgical approach (open, laparoscopic hand-assisted, pure laparoscopy, or robot-assisted). All variables were abstracted from institutional medical records by trained investigators following standardized registry definitions applied uniformly across all participating sites.

### 2.4. Statistical Analysis

Baseline clinical and pathological characteristics were summarized using descriptive statistics. Given the substantial imbalance in sample size and baseline features between HD and PD groups, propensity score overlap weighting was applied to estimate the average treatment effect in the overlap population (ATO). 

We calculated the propensity score using a logistic regression model:

$$log\left(\frac{\hat{e}(X_{i})}{1-\hat{e}(X_{i})}\right)=\beta_{0}+\beta_{1}X_{i1}+\beta_{2}X_{i2}+...+\beta_{p}X_{ip}$$

where $\hat{e}(X_{i})=\;P(ESRD_{i}=“1\;HD”|X_{i})$ is the probability of receiving hemodialysis. This model was calculated for all *i* = 1,2,…,350 where X*_i_* = {X*_i*1*_*,X*_i*2*_*,…X_*ip*_} represents the vector of baseline covariates *j* = 1,2,…,*p* (with *p* = 30 as the total number of covariates).

We then applied the overlap weighting formula to calculate individual weights (w*_i_*)

$$\omega_{i}=\left\{\begin{array}{c} 1-ê(Xi),\;\;\;\;\;{\mathrm{ESRD}}_{i}=“1\;HD”\\ ê(Xi),\;\;{\mathrm{ESRD}}_{i}=“2\;PD” \end{array}\right.\;$$

To ensure a valid assessment of overlap, we confirmed that all calculated propensity scores fell within the range of ê(X*_i_*)∈ (0,1).

We evaluated the balance of covariates before and after weighting using the Absolute Standardized Mean Difference (SMD). We calculated SMDs for continuous variables using the Equation

$$SMD=\frac{{\bar{X}}_{1}-{\bar{X}}_{2}}{\sqrt{(S_{1}^{2}+S_{2}^{2})/2}}$$

and for binary/categorical variables using Equation

$$SMD=\frac{p1-p2}{\sqrt{\left\{p1(1-p1)+p2(1-p2)\right\}/2}}$$

**Figure 2:**
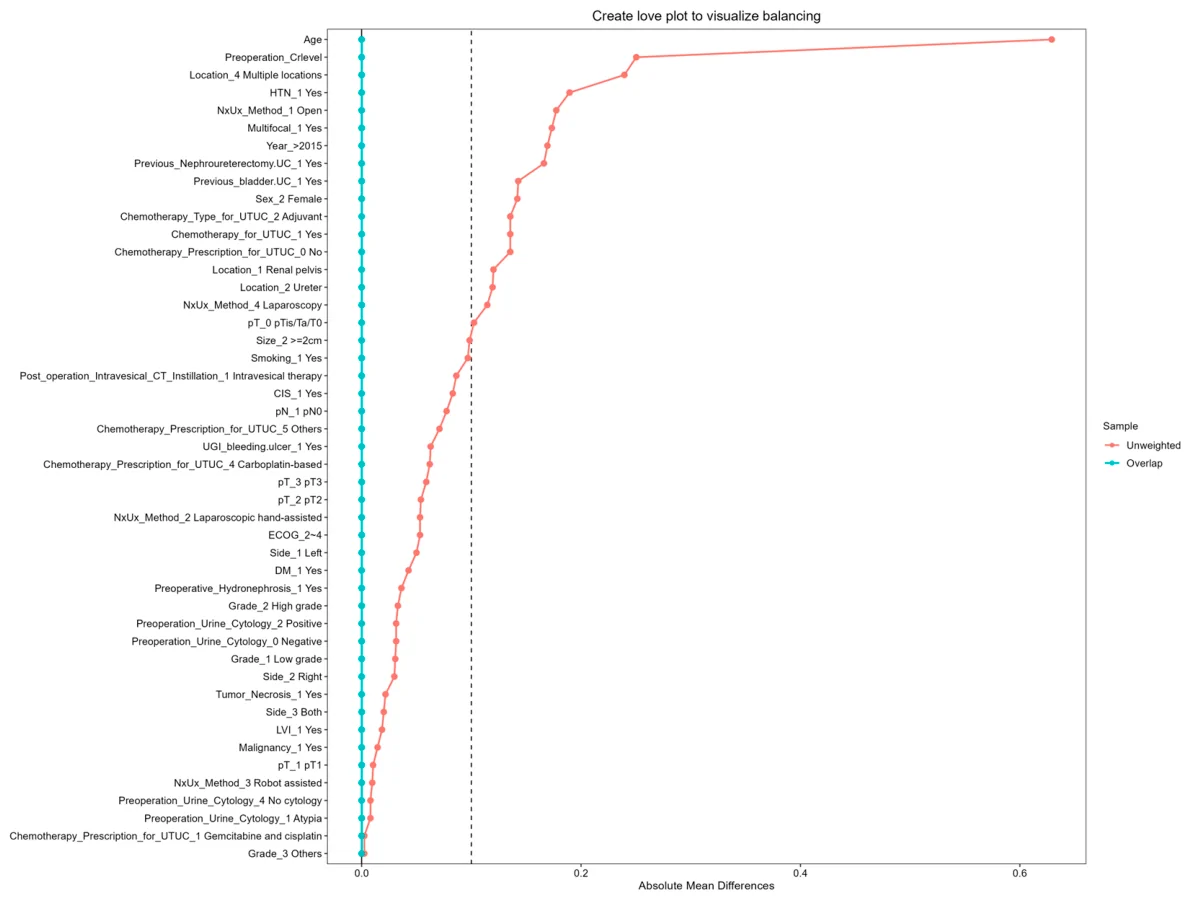
Love plot.

An SMD of less than 0.1 indicated that the groups were well-balanced. We used a Love plot to visualize this balance (Figure 2). 

To evaluate survival outcomes, we used:

Cox proportional hazards regression to analyze Overall Survival (OS) and standard Progression-Free Survival (PFS). We estimated survival curves using the Kaplan–Meier method and compared them using the log-rank test.

Fine-Gray subdistribution hazard models to analyze Cancer-Specific Survival (CSS), Time to Progression (TTP), and Non-UTUC mortality, treating competing deaths as competing risks. We estimated these probabilities using cumulative incidence functions.

All survival models used robust sandwich variance estimators to calculate standard errors, which adjusted our estimates for differences across the 21 participating hospitals (center adjustment). We also included the surgical era (Before 2015 versus 2015 and After) as an adjusted covariate in our multivariate models to control for changes in surgical techniques over time. Given the limited number of events in certain outcome categories (e.g., cancer-specific death), covariates included in each multivariable model were clinically prespecified a priori based on established prognostic relevance in UTUC, rather than selected through data-driven stepwise procedures, in order to minimize model instability and overfitting. All statistical tests were two-sided, and a *p*-value of less than 0.05 was considered statistically significant.

## 3. Results

### 3.1. Baseline Characteristics (Table 1)

A total of 350 ESRD patients undergoing RNU for UTUC were included: 310 on hemodialysis (HD) (88.6%) and 40 on peritoneal dialysis (PD) (11.4%) before radical nephroureterectomy (RNU). Before propensity score weighting, several significant baseline imbalances existed between the two groups (Table 1): PD patients were meaningfully younger (65.05 vs. 58.84 years; *p* < 0.001, SMD = 0.654), had higher rates of hypertension (72.5% vs. 53.5%; *p* = 0.036, SMD = 0.400), less rate of previous contralateral or ipsilateral upper tract urothelial carcinoma (UTUC) treated with nephroureterectomy (21.6% vs. 5.0%; *p* = 0.023, SMD = 0.504). Regarding tumor characteristics, PD patients had a higher proportion of multiple tumor locations (57.5% vs. 33.5%; *p* = 0.012, SMD = 0.496), while other pathological features were generally comparable between groups.

**Table 1:** Baseline Characteristics (*N* = 350).

Variables	Levels	1 HD	2 PD	*p*	SMD
***N***		**310**	**40**		
ECOG (%)	0~1	278 (89.7)	38 (95.0)	0.432	0.201
	2~4	32 (10.3)	2 (5.0)		
Sex (%)	1 Male	106 (34.2)	8 (20.0)	0.104	0.323
	2 Female	204 (65.8)	32 (80.0)		
Age (mean (SD))		65.05 (9.75)	58.84 (9.25)	<0.001 *	0.654
HTN (%)	0 No	144 (46.5)	11 (27.5)	0.036 *	0.400
	1 Yes	166 (53.5)	29 (72.5)		
DM (%)	0 No	258 (83.2)	35 (87.5)	0.644	0.121
	1 Yes	52 (16.8)	5 (12.5)		
Malignancy (%)	0 No	268 (86.5)	34 (85.0)	0.994	0.042
	1 Yes	42 (13.5)	6 (15.0)		
Side (%)	1 Left	155 (50.0)	22 (55.0)	0.748	0.136
	2 Right	141 (45.5)	17 (42.5)		
	3 Both	14 (4.5)	1 (2.5)		
Location (%)	1 Renal pelvis	107 (34.5)	9 (22.5)	0.012 *	0.496
	2 Ureter	99 (31.9)	8 (20.0)		
	4 Multiple locations	104 (33.5)	23 (57.5)		
Multifocal (%)	0 No	170 (54.8)	15 (37.5)	0.058	0.353
	1 Yes	140 (45.2)	25 (62.5)		
Size (%)	1 < 2 cm	139 (44.8)	14 (35.0)	0.312	0.202
	2 ≥ 2 cm	171 (55.2)	26 (65.0)		
pT (%)	0 pTis/Ta/T0	86 (27.7)	7 (17.5)	0.467	0.278
	1 pT1	104 (33.5)	13 (32.5)		
	2 pT2	53 (17.1)	9 (22.5)		
	3 pT3	67 (21.6)	11 (27.5)		
pN (%)	0 pNx	224 (72.3)	32 (80.0)	0.395	0.182
	1 pN0	86 (27.7)	8 (20.0)		
Grade (%)	1 Low grade	37 (11.9)	6 (15.0)	0.850	0.092
	2 High grade	266 (85.8)	33 (82.5)		
	3 Others	7 (2.3)	1 (2.5)		
Pre-operation Cr level (mean (SD))		7.47 (3.52)	8.50 (4.48)	0.094	0.254
NxUx Method (%)	1 Open	117 (37.7)	8 (20.0)	0.173	0.403
	2 Laparoscopic hand-assisted	61 (19.7)	10 (25.0)		
	3 Robot-assisted	28 (9.0)	4 (10.0)		
	4 Laparoscopy	104 (33.5)	18 (45.0)		
Smoking (%)	0 No	280 (90.3)	40 (100.0)	0.079	0.463
	1 Yes	30 (9.7)	0 (0.0)		
Previous Nephroureterectomy UC (%)	0 No	243 (78.4)	38 (95.0)	0.023 *	0.504
	1 Yes	67 (21.6)	2 (5.0)		
Previous bladder UC (%)	0 No	227 (73.2)	35 (87.5)	0.078	0.365
	1 Yes	83 (26.8)	5 (12.5)		
CIS (%)	0 No	204 (65.8)	23 (57.5)	0.390	0.171
	1 Yes	106 (34.2)	17 (42.5)		
Pre-operation Urine Cytology (%)	0 Negative	64 (20.6)	7 (17.5)	0.946	0.103
	1 Atypia	49 (15.8)	6 (15.0)		
	2 Positive	60 (19.4)	9 (22.5)		
	4 No cytology	137 (44.2)	18 (45.0)		
LVI (%)	0 No	277 (89.4)	35 (87.5)	0.932	0.058
	1 Yes	33 (10.6)	5 (12.5)		
Preoperative Hydronephrosis (%)	0 No	143 (46.1)	17 (42.5)	0.791	0.073
	1 Yes	167 (53.9)	23 (57.5)		
Tumor Necrosis (%)	0 No	280 (90.3)	37 (92.5)	0.876	0.078
	1 Yes	30 (9.7)	3 (7.5)		
Chemotherapy for UTUC (%)	0 No	290 (93.5)	32 (80.0)	0.008 *	0.408
	1 Yes	20 (6.5)	8 (20.0)		
Chemotherapy Type for UTUC (%)	0 No	290 (93.5)	32 (80.0)	0.008 *	0.408
	2 Adjuvant	20 (6.5)	8 (20.0)		
Chemotherapy Prescription for UTUC (%)	0 No	290 (93.5)	32 (80.0)	0.006 *	0.443
	1 Gemcitabine and cisplatin	7 (2.3)	1 (2.5)		
	4 Carboplatin-based	4 (1.3)	3 (7.5)		
	5 Others	9 (2.9)	4 (10.0)		
Post-operation Intravesical C/T Instillation (%)	0 No	298 (96.1)	35 (87.5)	0.046 *	0.319
	1 Intravesical therapy	12 (3.9)	5 (12.5)		
Year (%)	Before 2015	130 (41.9)	10 (25.0)	0.059	0.365
	After 2015	180 (58.1)	30 (75.0)		
Follow-up for OS/CSS		67.53 (42.84)	47.37 (35.65)	0.005 *	0.511
Follow-up for BRFS/DFS		45.61 (36.49)	32.70 (31.28)	0.033 *	0.380

* Statistically significant difference between HD and PD groups (*p* < 0.05). ECOG: Eastern Cooperative Oncology Group, HTN: Hypertension, DM: Diabetes Mellitus, UC: Urothelial carcinoma, CIS: Carcinoma in situ, LVI: Lymphovascular Invasion, UTUC: Upper tract urothelial carcinoma, C/T: Chemotherapy, OS: Overall survival, CSS: Cancer-specific survival, BRFS: Bladder recurrence-free survival, DFS: Disease-free survival, SMD: Standardized Mean Difference.

Of note, after excluding patients who received salvage or palliative treatments, PD patients received adjuvant chemotherapy more frequently than the HD group (20.0% vs. 6.5%; *p* = 0.008, SMD = 0.408) and postoperative intravesical chemotherapy instillation (12.5% vs. 3.9%; *p* = 0.046, SMD = 0.319). The follow-up duration for OS/CSS was shorter in the PD group (47.37 vs. 67.53 months, *p* = 0.005).

Other variables, such as ECOG performance status, sex, tumor size, pathological T stage, pathological N stage, histological grade, lymphovascular invasion (LVI), concomitant carcinoma in situ (CIS), preoperative creatinine level, and surgical approach, did not show statistically significant differences before weighting.

### 3.2. Propensity Score Overlap Weighting and Covariate Balance

To control for these baseline imbalances, we applied propensity score overlap weighting. This achieved a pseudo-sample size of 175.0 in both the HD and PD groups (Table 2). After overlap weighting, all 30 baseline covariates achieved an absolute standardized mean difference (SMD) and were successfully reduced below 0.1, indicating adequate balance between the two cohorts, as visually confirmed by the Love plot (Figure 2). The propensity score distributions before and after weighting are visualized in Figure S4, showing a substantial increase in overlap after weighting.

### 3.3. Perioperative and Oncological Recurrence Outcomes

The overlap-weighted perioperative outcomes and postoperative recurrence patterns are summarized in Table 2.

Perioperative complication severity (Clavien–Dindo classification) and postoperative bladder urothelial carcinoma did not differ significantly between groups (both *p* > 0.05). Similarly, intravesical recurrence, distant metastasis, disease-free status, and bladder recurrence rates were comparable between the HD and PD groups after weighting (all *p* > 0.05).

In contrast, the PD group demonstrated a significantly higher weighted rate of lower ureter/bladder cuff recurrence (5.3% vs. 0.4%, *p* = 0.001) and a significantly lower weighted rate of lymphatic metastasis (0.0% vs. 6.4%, *p* = 0.049) compared with the HD group; however, both event counts were derived from very small weighted cell sizes and should be interpreted with caution given the limited number of underlying PD patients (*n* = 40 unweighted). The PD group also showed significantly higher weighted overall mortality (53.0% vs. 30.6%, *p* = 0.025) and a significantly higher weighted rate of disease progression (53.0% vs. 32.9%, *p* = 0.046) than the HD group, consistent with the survival analyses described below. Cancer-specific mortality did not differ significantly between groups after weighting (13.5% vs. 10.3%, *p* = 0.631). Since these raw percentages do not account for survival time or competing risks, formal regression analyses were subsequently performed.

### 3.4. Multivariate Survival and Progression Analyses

#### Cancer-Specific Survival and Progression-Free Survival

**Figure 3:**
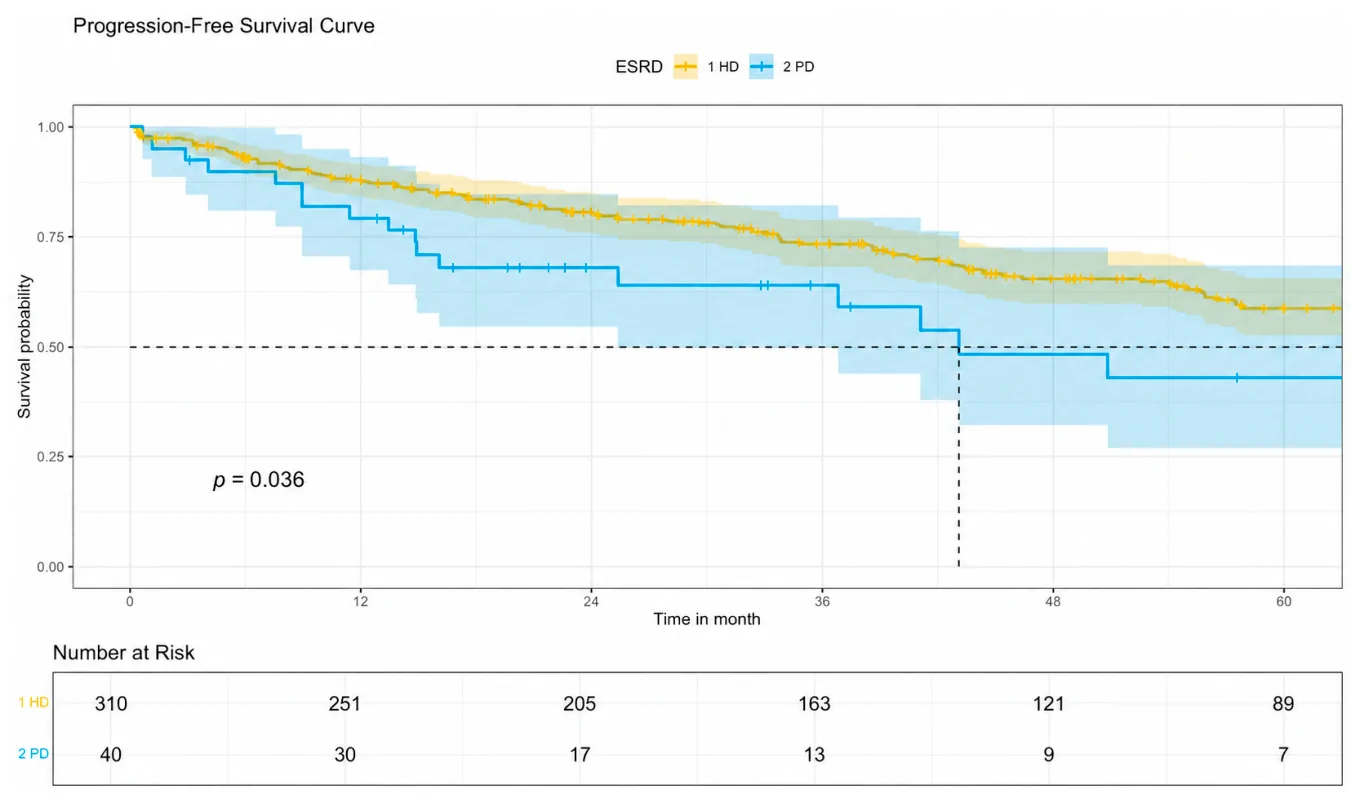
Progression-Free Survival Curve. Kaplan–Meier curve illustrates the progression-free survival (PFS) for patients on hemodialysis (HD, yellow line) versus peritoneal dialysis (PD, blue line) following propensity. The curve visually demonstrates a significantly inferior PFS in the PD cohort (*p* = 0.036), indicating a higher risk of disease progression. (Abbreviations: HD = hemodialysis; PD = peritoneal dialysis). The dashed line indicates the median survival time.

In the unweighted cohort, Kaplan–Meier curves for conventional progression-free survival (PFS), defined as progression or death from any cause, showed significantly worse survival for the PD group compared to the HD group (*p* = 0.036; Figure 3). In the multivariate Cox proportional hazards model (Table 3; univariate analyses are presented in Table S1), PD was independently associated with worse conventional PFS compared to HD (hazard ratio [HR] = 2.06, 95% confidence interval [CI]: 1.16–3.66; *p* = 0.013). Older age was also identified as an independent risk factor for worse PFS (HR = 1.07 per year, 95% CI: 1.03–1.10; *p* < 0.001; Table 3). 

To evaluate the pure oncological progression rate and account for the high rate of non-cancer deaths in this cohort, we conducted a further analysis using the Fine-Gray subdistribution hazard model for Time to Progression (TTP) (Table 4; univariate analyses are presented in Table S2). After treating death prior to progression as a competing risk, PD remained independently associated with a significantly higher risk of disease progression (subdistribution hazard ratio [sHR] = 2.68, 95% CI: 1.62–4.44; *p* < 0.001). In addition to dialysis modality, older age (sHR = 1.07 per year, 95% CI: 1.05–1.09; *p* < 0.001) and ECOG performance status 2–4 (sHR = 1.89, 95% CI: 1.20–2.97; *p* = 0.006) were independent risk factors for a shorter TTP (Table 4).

**Figure 4:**
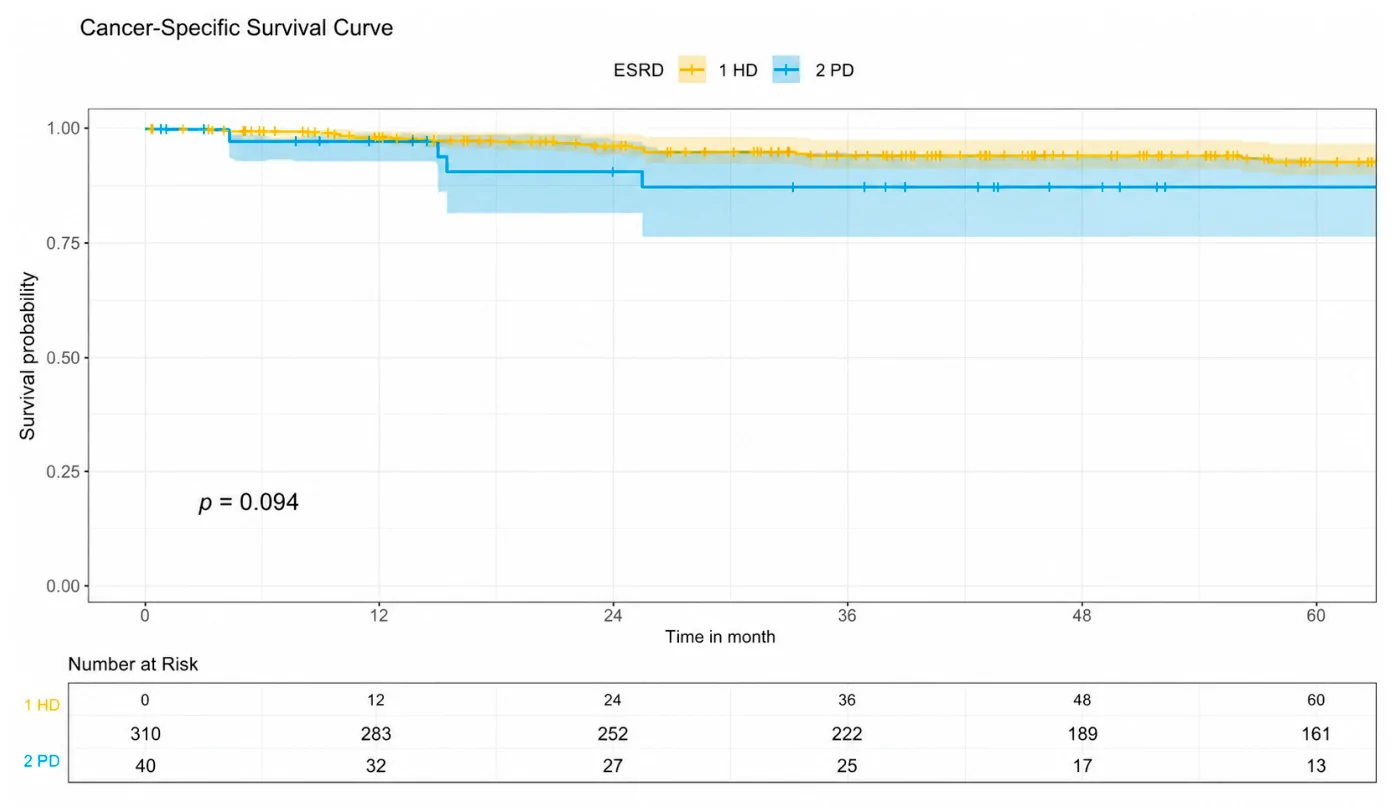
Cancer-Specific Survival Curve. Kaplan–Meier curve displays the cancer-specific survival (CSS) for the HD (yellow line) and PD (blue line) groups. A trend toward worse CSS is observed in the PD group (*p* = 0.094), with the survival curves showing consistent separation starting from approximately 12 to 24 months post-surgery onward. (Abbreviations: HD = hemodialysis; PD = peritoneal dialysis).

**Figure 5:**
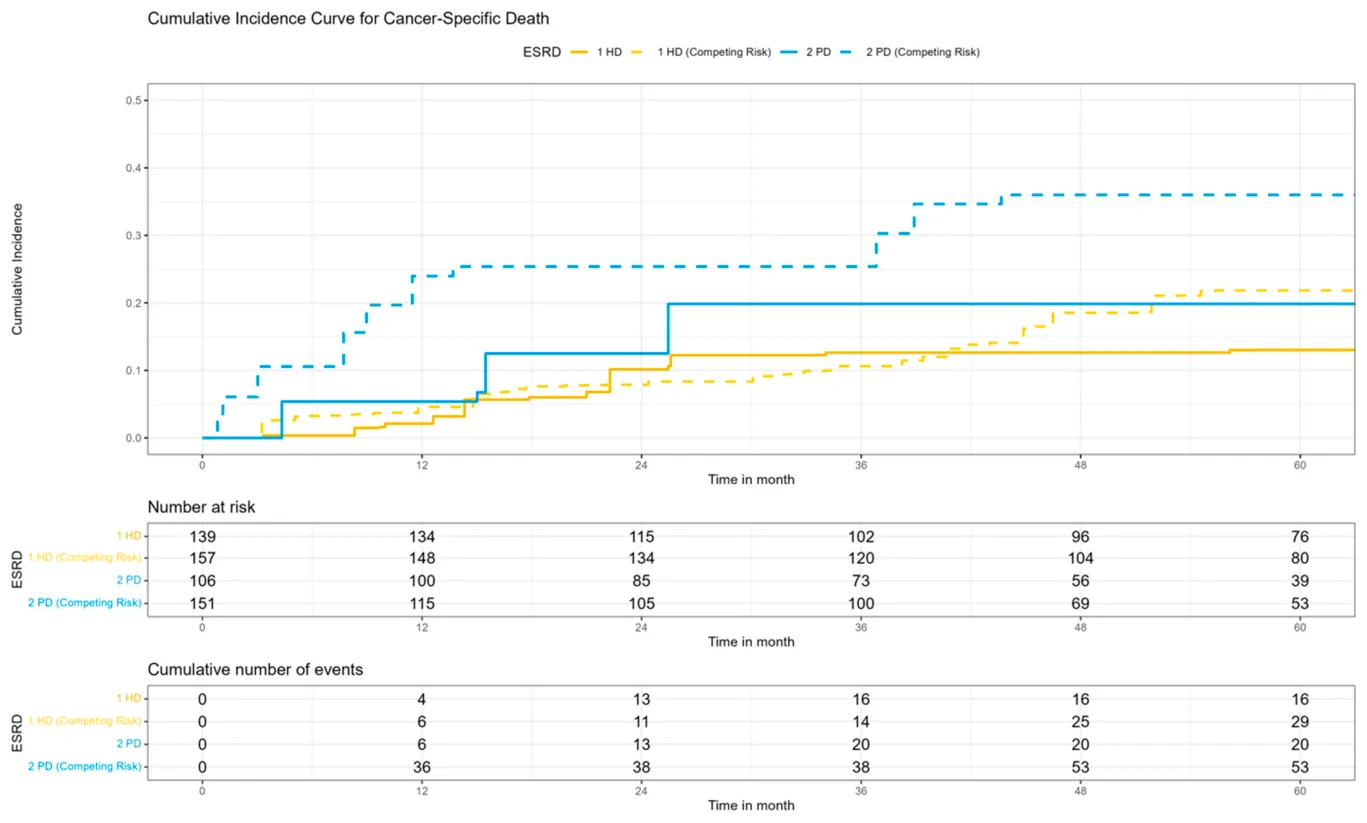
Cumulative incidence curves for Cancer-specific Death. The plot illustrates the cumulative incidence of cancer-specific mortality (solid lines) for the hemodialysis (HD, yellow) and peritoneal dialysis (PD, blue) groups, with non-cancer death treated as a competing risk (dashed lines). The cumulative incidence of cancer-specific mortality was not significantly different between the two dialysis groups after adjusting for covariates (multivariate subdistribution hazard ratio [sHR] = 1.71, 95% confidence interval [CI]: 0.64–4.56; *p* = 0.285 [Table 4]).

For cancer-specific survival (CSS), the Kaplan–Meier curves did not show a statistically significant difference between the two dialysis groups in the unweighted cohort (*p* = 0.094; Figure 4). In the multivariate Cox regression model (Table 3), PD was not significantly associated with worse CSS compared to HD (HR = 1.53, 95% CI: 0.45–5.14; *p* = 0.495). Similarly, in the multivariate Fine-Gray subdistribution hazard model (Table 4), treating non-cancer deaths as competing risks, dialysis modality was not significantly associated with CSS (sHR = 1.71, 95% CI: 0.64–4.56; *p* = 0.285). The cumulative incidence of cancer-specific death and its competing risk of non-cancer death are illustrated in Figure 5, which consistently demonstrated comparable cancer-specific mortality between the two dialysis cohorts under competing risk analysis. Instead, high tumor grade was identified as the primary independent predictor of worse CSS in the multivariate Cox model (HR = 9.96, 95% CI: 3.54–26.2; *p* < 0.001; Table 3).

**Figure 6:**
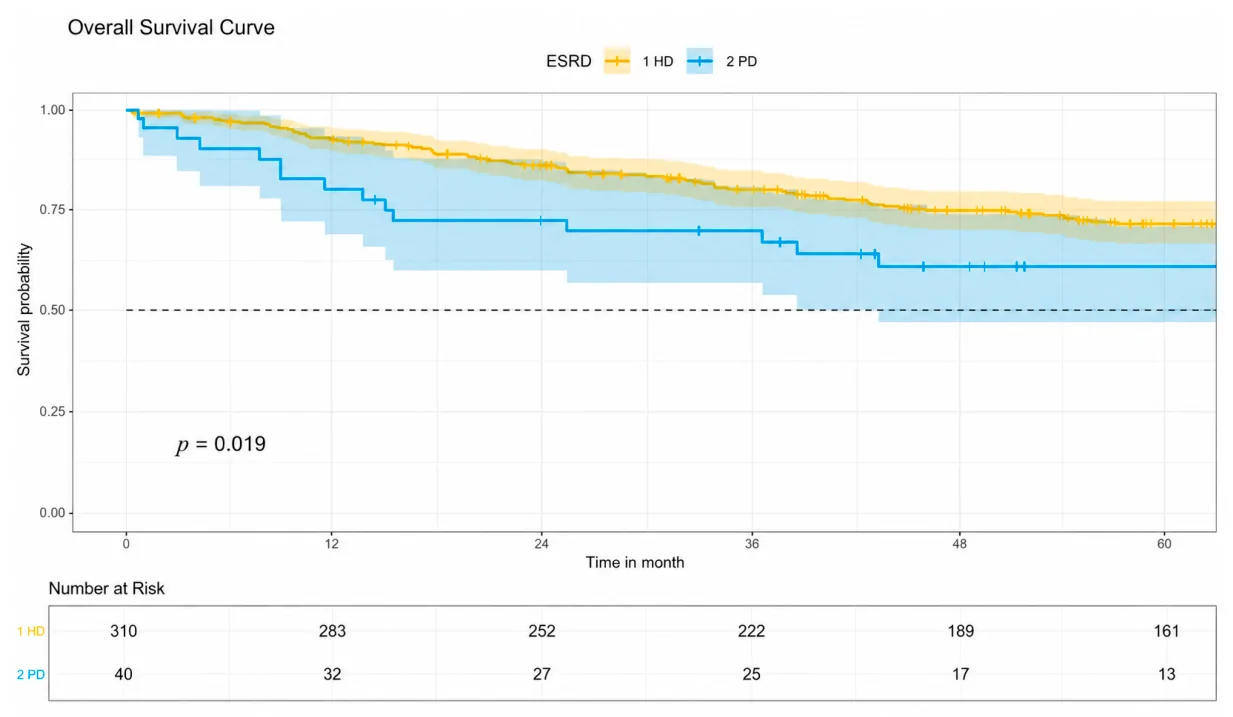
Overall Survival (OS) Curve. The survival curves illustrate the OS for end-stage renal disease (ESRD) patients following radical nephroureterectomy, stratified by baseline dialysis modality (PD vs. HD). *p*-value was calculated using the weighted log-rank test. (Abbreviations: HD = hemodialysis; PD = peritoneal dialysis; OS = overall survival; Non-UTUC Mortality). The dashed line indicates the median survival time.

In the multivariate Cox regression model (Table 3), PD remained an independent predictor of worse OS (HR = 2.34, 95% CI: 1.29–4.25; *p* = 0.005). Kaplan–Meier curves showed significantly worse overall survival (OS) for patients in the PD group compared to the HD group (*p* = 0.019; Figure 6) with PD patients reaching median OS at approximately 40 months while HD patients maintained >70% survival at 60 months. Additionally, the models identified older age (HR = 1.07 per year, 95% CI: 1.03–1.11; *p* = 0.001) and a history of smoking (HR = 3.86, 95% CI: 1.95–7.63; *p* < 0.001; Table 3).

**Figure 7:**
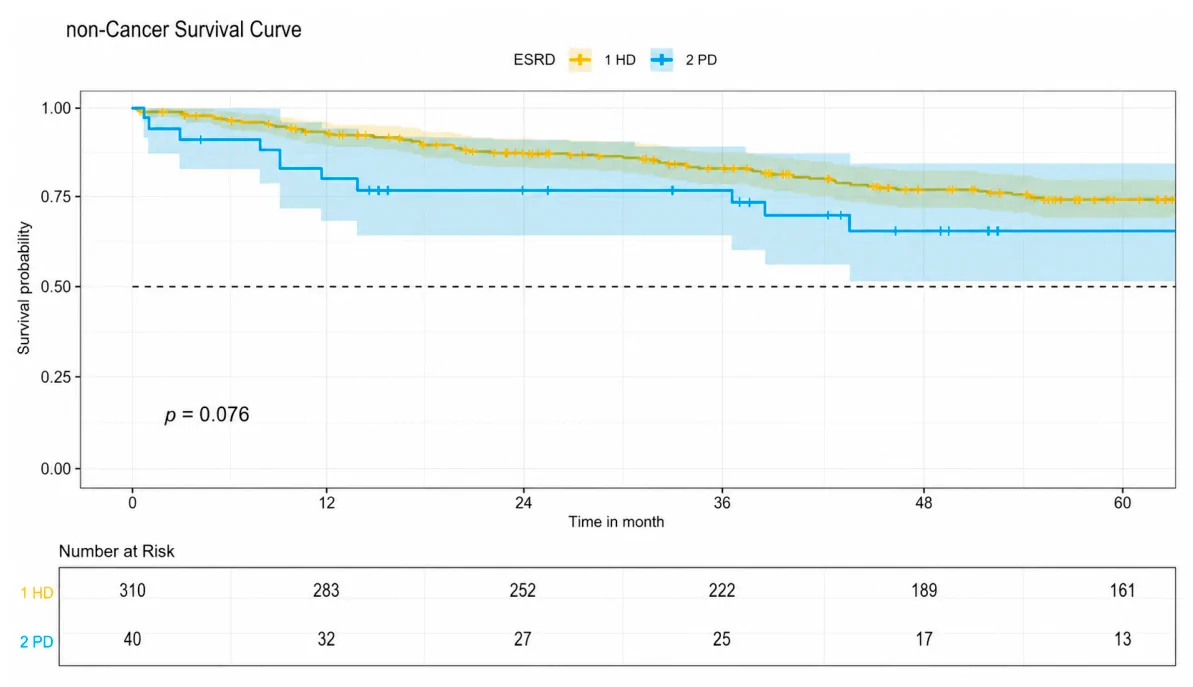
Non-Cancer Survival Curve. This Kaplan–Meier curve illustrates the non-cancer survival for patients on hemodialysis (HD, yellow line) versus peritoneal dialysis (PD, blue line) following propensity score overlap weighting. The curve visually supports the elevated non-cancer mortality risk in the PD cohort (*p* = 0.076), which is consistent with the significantly increased subdistribution hazard ratio observed in the multivariate Fine-Gray competing risk model.

In the analysis of non-UTUC mortality (non-cancer death), the unweighted Kaplan–Meier curves revealed a trend toward higher non-cancer survival in the HD group compared to the PD group (Log-rank *p* = 0.076; Figure 7). In the multivariate Cox regression analysis (Table 3), dialysis modality was not significantly associated with worse non-UTUC mortality (HR = 1.54, 95% CI: 0.48–4.91; *p* = 0.465).

**Figure 8:**
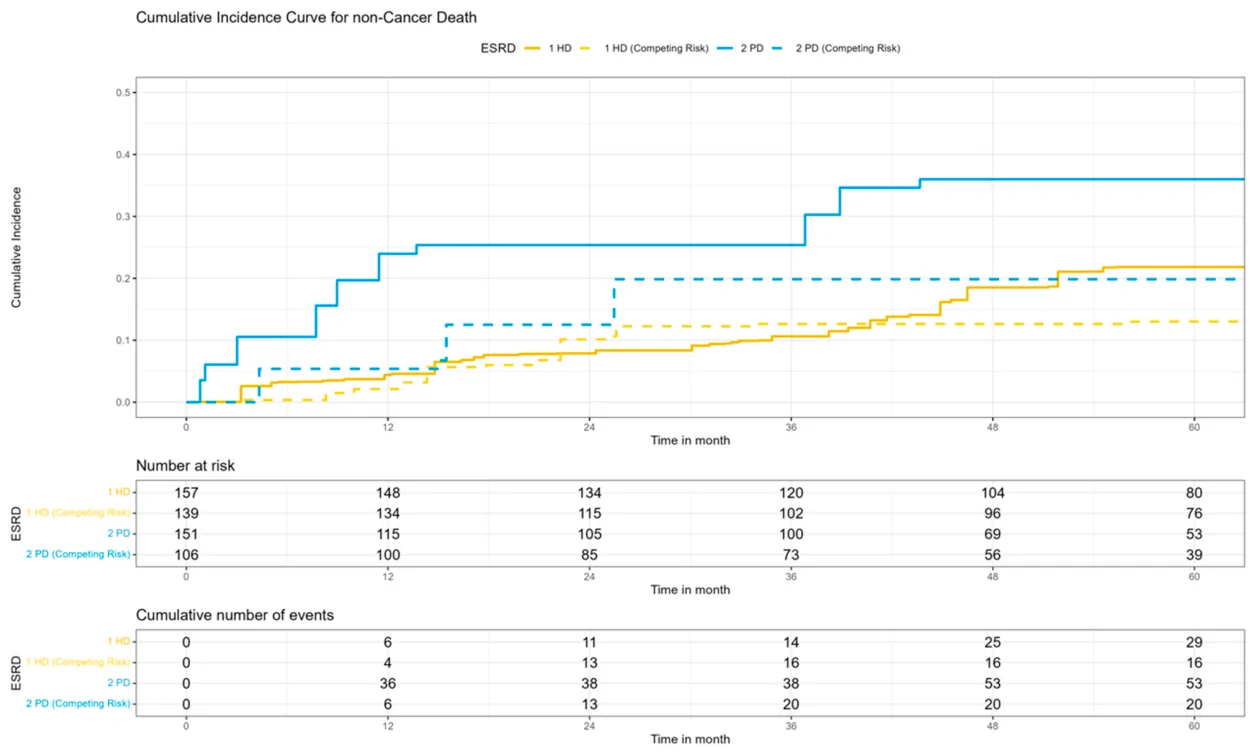
Cumulative incidence curves for non-cancer death. The plot illustrates the cumulative incidence of non-cancer mortality (solid lines) for the hemodialysis (HD, yellow) and peritoneal dialysis (PD, blue) groups, with cancer-specific death treated as a competing risk (dashed lines). Patients in the PD group exhibited a significantly higher cumulative incidence of non-cancer death compared to those in the HD group (multivariate subdistribution hazard ratio [sHR] = 2.17, 95% confidence interval [CI]: 1.22–3.88; *p* = 0.009 [Table 4]). The dashed line indicates the median survival time.

However, in the multivariate Fine-Gray subdistribution hazard model (Table 4) where death from UTUC was treated as a competing risk, PD was independently associated with a significantly higher risk of non-UTUC mortality (sHR = 2.17, 95% CI: 1.22–3.88; *p* = 0.009; Table 4). Consistently, the cumulative incidence curves for non-cancer mortality under the competing risk framework (Figure 8) demonstrated a significantly higher cumulative incidence of non-cancer death in the PD cohort over time. Other independent predictors of worse non-UTUC mortality in this competing risk model included ECOG performance status 2–4 (sHR = 1.89, 95% CI: 1.14–3.15; *p* = 0.014) and older age (sHR = 1.07 per year, 95% CI: 1.04–1.09; *p* < 0.001; Table 4).

## 4. Discussion

In this multicenter retrospective cohort study of 350 ESRD patients undergoing RNU for UTUC, we show that PD modality is an independent adverse prognostic factor for OS and PFS, and exhibits a clinical trend toward worse CSS (Figure 5), after confounder adjustment using propensity score overlap weighting. 

Previous large-scale studies—including a meta-analysis by Takano et al. (2023) [14] and a nationwide Japanese cohort by Miyamoto et al. (2022) [13]—have clearly shown that dialysis patients experience worse postoperative outcomes after cancer surgeries. However, these studies only analyzed all dialysis patients as one single group. By not separating PD from hemodialysis (HD), the different clinical impacts of the two dialysis modalities remain unclear.

Even when previous population-based studies attempted to separate the modalities, such as Cherng et al. (2013) in their analysis of non-cardiac surgeries, they mixed extra-abdominal procedures (e.g., orthopedic or vascular) with major intra-abdominal surgeries [15]. Cherng et al. reported significantly higher postoperative mortality in both hemodialysis (OR 3.42, 95% CI 2.62–4.47) and peritoneal dialysis patients (OR 2.71, 95% CI 1.70–4.31), along with increased rates of myocardial infarction, pneumonia, bleeding, and septicemia, with risks further amplified by concurrent hypertension, diabetes, and additional comorbidities. 

Notably, when dialysis modality has been examined within a single, more homogeneous surgical category, the direction of risk can differ. In coronary artery bypass grafting, Li et al. (2017) found that PD patients had higher in-hospital mortality than HD patients (58.3% vs. 14.8%, *p* < 0.001) and lower 1-year survival (33.3% vs. 56.6%, *p* = 0.031), with septic shock rather than cardiac events driving PD mortality [16]. In contrast, a matched cohort study of cardiothoracic surgery by Kumar et al. (2011) reported no difference in operative mortality or 2-year survival between PD and HD patients, and even a lower rate of postoperative complications in the PD group (28% vs. 50%, *p* = 0.046) [17]. These conflicting findings from a single, relatively uniform surgical category further underscore that pooling PD and HD together—or generalizing findings across surgical types—may obscure clinically meaningful, modality-specific and procedure-specific differences.

This raises the question of what happens when the surgical field itself directly involves the peritoneal cavity, as is the case for RNU. Unlike cardiac or extra-abdominal procedures, RNU may directly disrupt the peritoneal cavity or affect peritoneal dialysis function, exposing PD patients to unique risks such as severe adhesions, dialysate leakage, and compromised dialysis adequacy, because their peritoneal environment is already fragile. To our knowledge, the current study represents the first systematic comparison of oncologic outcomes and non-oncologic outcomes between PD and HD patients following RNU for UTUC. 

Crucially, our subgroup analysis stratified by surgical era provides novel insights into this disparity. We observed that the elevated mortality and progression risks associated with PD were highly prominent in the earlier surgical era (before 2015) but substantially attenuated in contemporary practice (2015 and after) (Supplementary Figures S1–S3). This attenuation may be associated with modern advancements in perioperative care, improved dialysis catheter management, and the increasing adoption of minimally invasive or retroperitoneal surgical approaches, which minimize peritoneal cavity disruption. Nevertheless, even in the contemporary era, PD patients still exhibit a clinically meaningful trend toward worse OS and PFS (HRs > 1.7). This underscores that while modern surgical techniques can mitigate the survival disadvantage, PD remains a highly vulnerable state requiring meticulous, modality-specific perioperative management.

Potential mechanisms for worse survival in PD patients

We hypothesize that the survival disadvantage in PD may reflect two potential mechanisms: impaired systemic tumor control and elevated non-UTUC mortality. 

First: PD-Specific Immune Dysfunction and Impaired Systemic Tumor Control

Betjes et al. have demonstrated that patients with ESRD exhibit significant immune dysfunction, including increased susceptibility to infections and malignancies [18]. Superimposed on this baseline immune impairment, PD induces additional modality-specific immune alterations that further compromise anti-tumor surveillance, including reduced monocyte synthesis of TNF-α and IL-1β and inhibited T helper phenotype development compared with HD patients [19], impaired T cell cytokine production potentially indicating Th1 cell exhaustion [20], and the depletion of homeostatic tissue-resident peritoneal macrophages [21].

Importantly, the clinical impact of these immune alterations is amplified by their timing. The perioperative month has been recognized as a “vulnerable window” where surgery-induced stress creates a permissive environment for residual tumor cells to proliferate. Pan and Wang further demonstrated that immune recovery failure at postoperative day 30 was associated with greater molecular residual disease positivity and worse disease-free survival in colorectal cancer patients [22].

This concept of perioperative molecular residual disease (MRD) is not limited to colorectal cancer and is increasingly supported by disease-specific evidence within urothelial carcinoma itself. Nakano et al. demonstrated that in patients with localized UTUC undergoing radical nephroureterectomy, a preoperative circulating tumor DNA (ctDNA) fraction > 2% was an independent risk factor for worse recurrence-free survival, and persistent ctDNA positivity in the early postoperative period—consistent with MRD—was significantly associated with subsequent relapse [23]. Similarly, Tamura et al. prospectively monitored individualized ctDNA in plasma and urine after RNU and found that case-specific ctDNA became detectable at the time of intravesical recurrence or distant metastasis, with a mean lead time of 60 days for urinary ctDNA ahead of clinical recurrence [24]. Pierconti et al. showed that urine DNA methylation can detect cancer cells before they are visible on standard biopsies, though their reported 42% recurrence rate indicates a high rate of false positives [25].

These findings indicate that failure to clear tumor-derived molecular signal in the early postoperative period reflects biologically active residual disease in UTUC specifically, reinforcing the plausibility that impaired postoperative immune recovery—as may occur in PD patients—could translate into a higher burden of MRD and, consequently, worse oncologic control.

Within this critical timeframe, Caprara et al. reported that PD significantly increases circulating regulatory T cells (Tregs) [26] . While Treg accumulation may serve a beneficial role in attenuating uremic inflammation, Kumar et al. cautioned in their comprehensive review that the balance between Tregs and effector T cells is critical: excessive Treg levels can reflect a state of immune anergy and paralysis analogous to that observed in solid tumors [27]. Najaf et al. noticed that in the tumor microenvironment, an abundance of Tregs suppresses anti-tumor immune responses, allowing tumors to evade immune surveillance and promote cancer progression [28]. These findings collectively suggest that PD-induced Treg upregulation may shift the immune balance toward tumor-permissive conditions. While current evidence primarily captures short-term immune changes post-dialysis, this mechanism provides a plausible hypothesis for the poorer oncological control (worse PFS) observed in the PD cohort. However, no current study has demonstrated a direct causal link between PD-related immune modulation and UTUC outcomes and importantly, because we did not directly measure immune subsets (e.g., Tregs or peritoneal macrophages) in our registry, this mechanistic discussion remains strictly hypothesis-generating and further longitudinal investigations are warranted to validate this hypothesis.

Second: Surgery-Induced Peritoneal Disruption and Non-UTUC Mortality

Abdominal surgery causes unique challenges for PD patients. Lew and Collins highlighted that PD patients undergoing abdominal surgery were found to have an increased risk of infection, bleeding, and dialysate leakage, complications that may affect the future use of PD and, in certain cases, necessitate temporary or permanent transition to HD [29]. Cheng et al. demonstrated that patients with prior abdominal surgery had significantly higher rates of intra-abdominal adhesions, which were associated with reduced dialysis adequacy indices [30]. Qureshi et al. (2024), in a multicenter study, confirmed that intra-abdominal adhesions are associated with a higher risk of PD catheter-related complications (adjusted HR 1.64), including flow restriction, abdominal pain, need for invasive intervention, or PD termination [31]. RNU, as a major retroperitoneal or intraperitoneal procedure, would be expected to produce similar adhesive consequences, potentially compromising dialysis adequacy. 

While early observational data, including the CANUSA study, suggested a dose-dependent association between peritoneal Kt/V and patient survival [32], the absolute benefit of continuously escalating Kt/V targets has been challenged by subsequent prospective evidence. The ADEMEX trial demonstrated no survival advantage when peritoneal Kt/V was increased from approximately 1.6 to 2.27 in patients with residual renal function [33] and similar findings were observed by Szeto et al. and Fried et al. in anuric cohorts, where the survival benefit of higher peritoneal clearance appeared to plateau beyond a minimum threshold [34,35]. Regardless, inadequate peritoneal clearance is universally recognized to trigger fluid overload and adverse cardiovascular events. These findings provide a plausible mechanistic link between surgery-induced PD inadequacy and the steep early rise in non-cancer competing risk observed in our PD cohort (Table 4, Figure 8).

In addition, frailty is highly prevalent among dialysis patients and worsens over time on dialysis [36]. A meta-analysis showed that frail patients with cancer have significantly increased risks of mortality (HR 1.68), treatment-related toxicity (OR 1.83), and treatment intolerance (OR 1.68) [37]. 

Taken together, these data might suggest a vicious cycle in PD patients following RNU: surgical disruption of the peritoneal cavity compromises dialysis adequacy, which accelerates frailty and functional decline. When combined with their inherently poorer systemic tumor control (as evidenced by significantly worse PFS), PD patients may be less able to tolerate subsequent salvage therapies upon tumor recurrence. This profound vulnerability ultimately translates into the cancer-specific mortality risk (multivariate HR = 1.53, *p* = 0.495; sHR = 1.71, *p* = 0.285) observed in our refined cohort, running parallel to their elevated non-cancer mortality risk.

### 4.1. Influence of Patient Demographics and Chemotherapy

In routine nephrology practice, PD is conventionally favored for younger, functionally independent patients without a history of major abdominal surgery, primarily to preserve autonomy and quality of life. Previous literature, such as Stack (2002) and Chiang et al. (2016), has robustly demonstrated that younger age, higher educational levels, and greater functional status are independent predictors for selecting PD [38,39]. Consistent with this established selection bias, the PD patients in our cohort possessed a favorable baseline profile: they were significantly younger than the HD group (mean age 58.8 vs. 65.1 years) and received adjuvant chemotherapy more frequently (20.0% vs. 6.5%). 

However, despite these superior baseline characteristics and higher rates of systemic treatment, PD patients still showed significantly worse OS, PFS, and a strong trend toward worse CSS after RNU in our study. This paradox supports the hypothesis that the survival disadvantage of PD is probably not due to patient-inherent risk factors but due to the profound physiological consequences of undergoing major abdominal surgery within a severely compromised peritoneal environment. 

The paradox of worse outcomes despite higher chemotherapy utilization in PD patients may also be partly explained by altered drug pharmacokinetics. A systematic review by Labaki et al. (2020) found that data on anti-neoplastic agents in PD patients are extremely sparse, with multiple reports of altered drug elimination and serious adverse events [40]. Eads et al. (2016) demonstrated that cisplatin clearance was reduced in a PD patient, with negligible peritoneal dialysate contribution to drug elimination [41]. The pharmacokinetic alterations raise the possibility that chemotherapy efficacy may be suboptimal in PD patients, even when administered at standard doses, potentially contributing to the observed survival disadvantage.

### 4.2. Clinical Implication

Current international frameworks—most notably the 2025 European Association of Urology (EAU) guidelines [9] which underwent a complete revision of their risk stratification chapter, and the American Urological Association (AUA)/Society of Urologic Oncology guidelines [10] which stratify patients into low- and high-risk categories based on endoscopic, cytologic, pathologic, and radiographic findings—provide comprehensive, risk-adapted recommendations for the management of UTUC. However, these guidelines currently place limited emphasis on the specific postoperative management of dialysis-dependent patients, often treating ESRD as a general surgical risk factor rather than a condition requiring modality-specific surveillance. This lack of guidance for patients on peritoneal dialysis (PD) versus hemodialysis (HD) highlights a significant gap in our current clinical framework. 

Given the profound physiological consequences and elevated non-cancer mortality associated with standard RNU in this vulnerable population, the potential for kidney-sparing surgery (e.g., endoscopic management) should be highlighted. Although preserving renal function is no longer applicable to this ESRD cohort, kidney-sparing approaches may offer a plausible “major-surgery-sparing” alternative. For frail, high-risk ESRD patients who meet appropriate oncological criteria, these approaches can minimize surgical trauma, avoid severe peritoneal disruption, and reduce the risk of catastrophic postoperative decompensation.

In the preoperative setting, baseline dialysis adequacy, including dialysis adequacy indices (weekly Kt/V urea) and peritoneal membrane transport characteristics assessed by peritoneal equilibration test (PET), should be formally assessed, and documented prior to RNU. A multidisciplinary discussion involving urology and nephrology is strongly recommended to determine whether temporary bridging to hemodialysis should be initiated before surgery, particularly in patients with borderline dialysis adequacy or prior abdominal adhesions that may compromise postoperative PD function.

In the postoperative setting, the perioperative and surveillance phases should be approached as a unified, phased protocol that takes both dialysis-related complications and oncological risk simultaneously into consideration. The postoperative period represents a critical window of vulnerability, during which disruption of the peritoneal environment may precipitate dialysis inadequacy, heightened infectious risk, and cardiovascular decompensation [29,42]. Furthermore, our survival data demonstrate that the PFS curve between PD and HD patients begins to diverge significantly at 12–24 months post-surgery (Figure 3).

Although the small sample size of our PD cohort prevents us from making definitive guidelines, we suggest the following practical, phased surveillance plan as a hypothesis-driven strategy requiring future prospective validation:

Phase 1 (Months 0–6): This phase addresses the immediate surgical impact on peritoneal integrity and dialysis function. Feasibility of resuming PD should be reassessed within 48–72 h of surgery. For small-incision or laparoscopic procedures especially via a retroperitoneal approach, PD can often be resumed within 48 h using small-volume exchanges in the supine position; however, for procedures involving large anterior abdominal incisions, such as open RNU or transperitoneal laparoscopic approaches, a 2-to-3-week hiatus from PD is generally recommended [42]. Given the high risks of dialysate leakage and catheter dysfunction after RNU, we recommend a prompt, temporary switch to hemodialysis (HD) if PD resumption is complicated. Once PD is safely resumed, alongside routine oncological care, close monthly monitoring of dialysis adequacy (weekly Kt/V), nutrition, and infection markers is essential.

Phase 2 (months 6–24): This phase corresponds to the interval of maximal oncological risk divergence observed in our cohort. Current AUA/SUO guidelines recommend cross-sectional imaging of the abdomen and pelvis every 3–6 months for the first 2 years after RNU for ≥pT2 disease, every 6 months at year 3, and annually thereafter to year 5, with chest imaging every 6–12 months for the first 5 years [10]. Given the heightened oncological survival risk observed in PD patients in our study, more intensive follow-up with shorter intervals should be considered. However, frequent invasive examinations can be rather difficult for frail dialysis patients to cope with. As reviewed by Ricciardi et al., emerging non-invasive biomarkers, including urinary DNA methylation, may in the future complement standard imaging to enable earlier, less invasive detection of recurrence [43]. Although these epigenetic tools are not yet mature enough to replace standard endoscopy, they could be used alongside standard imaging to monitor high-risk patients. 

Ongoing regular assessment of dialysis adequacy should be maintained in parallel, given that systemic deterioration and tumor recurrence may co-occur and mutually accelerate during this critical window.

Due to the dual burden of oncological and non-oncological risks in this population, a formal multidisciplinary team (MDT)—integrating urology, nephrology, and oncology—is highly recommended. Such an integrated framework allows for coordinated decision-making across all clinical phases and provides tailored strategies to mitigate the specific risks of each dialysis modality.

## 5. Limitations

Several limitations should be noted. First, despite being the largest cohort of its kind to date, the sample size of the PD group remains limited (*n* = 40). This restricted our statistical power to perform more complex time-varying survival analyses and resulted in wide confidence intervals in certain specific subgroups (e.g., high-grade tumors). In particular, the limited number of cancer-specific death events resulted in wide confidence intervals for some covariates in the CSS model, which should be interpreted with appropriate caution. Furthermore, while propensity score overlap weighting successfully maximized covariate balance, it inherently estimates the average treatment effect for the overlap population—a “pseudo-population” of patients who share characteristics making them eligible for either modality. Therefore, our findings may have limited generalizability to patients with absolute contraindications to either HD or PD. 

Second, our survival endpoints relied on the Taiwan Ministry of Health and Welfare (MOHW) cause-of-death registry. While this national registry is highly reliable for distinguishing cancer from non-cancer mortality, it lacks detailed information on the exact causes of non-UTUC deaths. Consequently, our mechanistic discussion regarding the exact specific causes of the elevated non-cancer competing risk in PD patients remains exploratory. 

Third, as a retrospective registry study, our dataset lacked certain granular operative details, such as the specific techniques used for bladder cuff excision (e.g., open, extravesical, or endoscopic approaches). Furthermore, we were unable to track long-term changes in dialysis adequacy and immune function after surgery, nor do we have the exact dates of modality switches to perform time-dependent analyses. We also lacked data on the exact duration of dialysis dependence prior to RNU. The absence of these longitudinal variables prevents us from establishing a definitive causal relationship between RNU-induced peritoneal disruption and subsequent mortality.

Finally, to strictly adhere to RECIST 1.1 criteria for defining disease progression, our inclusion criteria necessitated at least one postoperative imaging assessment. While this requirement ensured oncological rigor, it may introduce a degree of selection bias, as highly frail patients who died very early before their first scheduled imaging scan might have been inadvertently excluded. Additionally, the shorter median follow-up duration in the PD group (47.4 vs. 67.5 months), while likely reflecting the higher and earlier incidence of competing mortality in these patients, limits our ability to confidently predict survival outcomes beyond five years. Although rigorous overlap weighting was applied, unmeasured residual confounding inherent to observational designs cannot be entirely ruled out. Specifically, we lacked data on residual renal function, nutritional status (e.g., serum albumin), frailty, vascular access type, and specific comorbidity severity, which may further influence modality selection and survival. Dialysis modality selection is heavily influenced by physician-patient shared decision-making, which cannot be perfectly captured by retrospective datasets.

## 6. Conclusions

In this multicenter study, peritoneal dialysis was independently associated with significantly worse overall and progression-free survival and exhibited a trend toward worse cancer-specific survival in univariate analysis. This survival disadvantage persisted despite the PD group being younger and receiving more adjuvant chemotherapy. We hypothesize that these inferior outcomes may be driven by a dual burden of both weaker systemic tumor control and an increased risk of non-UTUC mortality after surgery. To minimize these risks, we propose, as a hypothesis-generating strategy requiring prospective validation, prioritizing minimally invasive or retroperitoneal surgical approaches to preserve peritoneal integrity whenever feasible, coupled with rigorous, modality-specific multidisciplinary surveillance to optimize postoperative dialysis adequacy.

## Figures and Tables

**Table 2:** Outcome Variables Baseline (overlap weighted).

Variables	Levels	Unweighted			Overlap Weighted		
		1 HD	2 PD	*p*	1 HD	2 PD	*p*
*N*		310	40		175.0	175.0	
Clavien–Dindo Classification (%)	0 No Complication	158 (51.0)	22 (55.0)	0.863	105.7 (60.4)	96.0 (54.9)	0.222
	1 Grade I/II	138 (44.5)	16 (40.0)		66.8 (38.2)	65.7 (37.5)	
	2 Grade III/IV	14 (4.5)	2 (5.0)		2.4 (1.4)	13.3 (7.6)	
Post-operation Bladder UC (%)	0 No	229 (73.9)	25 (62.5)	0.184	120.2 (68.7)	118.0 (67.4)	0.894
	1 Yes	81 (26.1)	15 (37.5)		54.8 (31.3)	57.0 (32.6)	
Lower Ureter or Bladder Cuff Recurrence (%)	0 No	306 (98.7)	38 (95.0)	0.292	174.3 (99.6)	165.7 (94.7)	0.001 *
	1 Yes	4 (1.3)	2 (5.0)		0.7 (0.4)	9.3 (5.3)	
Lymphatic Metastasis (%)	0 No	295 (95.2)	40 (100.0)	0.314	163.9 (93.6)	175.0 (100.0)	0.049 *
	1 Yes	15 (4.8)	0 (0.0)		11.1 (6.4)	0.0 (0.0)	
Distant Metastasis (%)	0 No	290 (93.5)	36 (90.0)	0.615	156.1 (89.2)	157.3 (89.9)	0.920
	1 Yes	20 (6.5)	4 (10.0)		18.9 (10.8)	17.7 (10.1)	
Overall Mortality (%)	0 No	194 (62.6)	21 (52.5)	0.289	121.4 (69.4)	82.2 (47.0)	0.025 *
	1 Yes	116 (37.4)	19 (47.5)		53.6 (30.6)	92.8 (53.0)	
Cancer-specific (%)	0 No	288 (92.9)	35 (87.5)	0.373	157.0 (89.7)	151.3 (86.5)	0.631
	1 Yes	22 (7.1)	5 (12.5)		18.0 (10.3)	23.7 (13.5)	
Intravesical recurrence (%)	0 No	243 (78.4)	28 (70.0)	0.321	130.9 (74.8)	130.8 (74.7)	0.993
	1 Yes	67 (21.6)	12 (30.0)		44.1 (25.2)	44.2 (25.3)	
Progression-Free (%)	0 No	121 (39.0)	19 (47.5)	0.391	57.5 (32.9)	92.8 (53.0)	0.046 *
	1 Yes	189 (61.0)	21 (52.5)		117.5 (67.1)	82.2 (47.0)	
Disease-Free (%)	0 No	32 (10.3)	5 (12.5)	0.882	26.6 (15.2)	19.9 (11.4)	0.593
	1 Yes	278 (89.7)	35 (87.5)		148.4 (84.8)	155.1 (88.6)	
Bladder Recurrence (%)	0 No	229 (73.9)	25 (62.5)	0.184	120.2 (68.7)	118.0 (67.4)	0.894
	1 Yes	81 (26.1)	15 (37.5)		54.8 (31.3)	57.0 (32.6)	

* Indicates statistical significance (*p* < 0.05). Abbreviation: UC: Urothelial carcinoma.

**Table 3:** Multivariate Cox Regression for Overall Survival (OS), Cancer-Specific Survival (CSS), non-cancer-specific survival (nCS), Progression-Free Survival (PFS).

Variables	Levels	OS		CSS		nCS (Non-Cancer)		PFS	
		HR	*p*	HR	*p*	HR	*p*	HR	*p*
ESRD	1 HD	1		1		1		1	
	2 PD	2.34 (1.29, 4.25)	0.005 *	1.53 (0.45, 5.14)	0.495	1.54 (0.48, 4.91)	0.465	2.06 (1.16, 3.66)	0.013 *
Age	Mean (SD)	1.07 (1.03, 1.11)	0.001 *					1.07 (1.03, 1.10)	<0.001 *
Grade	1 Low grade			1					
	2 High grade			9.96 (3.54, 26.2)	<0.001 *				
Smoking	0 No	1							
	1 Yes	3.86 (1.95, 7.63)	<0.001 *						

* Indicates statistical significance (*p* < 0.05). Abbreviations: OS = overall survival; CSS = cancer-specific survival; PFS = progression-free survival; HR = hazard ratio; ESRD = end-stage renal disease; HD = hemodialysis; PD = peritoneal dialysis.

**Table 4:** Multivariate Fine-Gray Hazard Model for Cancer-Specific Survival (CSS), non-cancer-specific survival (nCS), Time-to-progression (TTP).

Variables	Levels	CSS		nCS (Non-Cancer)		TTP	
		HR	*p*	HR	*p*	HR	*p*
ESRD	1 HD	1		1		1	
	2 PD	1.71 (0.64, 4.56)	0.285	2.17 (1.22, 3.88)	0.009 *	2.68 (1.62, 4.44)	<0.001 *
ECOG	0~1			1		1	
	2~4			1.89 (1.14, 3.15)	0.014 *	1.89 (1.20, 2.97)	0.006 *
Age	Mean (SD)			1.07 (1.04, 1.09)	<0.001 *	1.07 (1.05, 1.09)	<0.001 *
RNU Method	1 Open	1					
	2 LPS hand-assisted	2.67 (1.03, 6.94)	0.044 *				
	3 Robot-assisted	0.62 (0.08, 5.09)	0.659				
	4 Laparoscopy	1.22 (0.44, 3.39)	0.705				

In the Fine-Gray competing risk analysis, non-UTUC mortality was treated as the competing event for cancer-specific survival (CSS). For time-to-progression (TTP), death prior to disease progression was treated as the competing event. Abbreviations: HD = hemodialysis; PD = peritoneal dialysis; HR = hazard ratio; ECOG = Eastern Cooperative Oncology Group; Overall Survival. * Indicates statistical significance (*p* < 0.05).

## Data Availability

The data associated with this study are available from the corresponding author upon reasonable request.

## Supplementary Materials

The following supporting information can be downloaded at https://www.mdpi.com/article/10.3390/cancers18162670/s1. Figure S1: Progression-Free Survival Model with Subgroups Stratified by Surgical Era (Overlap weighted); Figure S2: Cancer-Specific Survival Model with Subgroups Stratified by Surgical Era (Overlap weighted); Figure S3: Overall Survival Model with Subgroups Stratified by Surgical Era (Overlap weighted); Figure S4: Propensity score distributions before and after overlap weighting; Table S1: Univariate Model Cox Regression for Survival; Table S2: Univariate Fine-Gray Hazard Model for Survival.
