# Supplementary material for: Does Dialysis Type Matter? Re-Evaluating Prognosis in UTUC Patients Following Surgery
Source: Cancers (Basel). 2026 Aug 18;18(16):2670. doi: 10.3390/cancers18162670 (PMC13510789; doi:10.3390/cancers18162670)
Supplement: Supplementary file 1 [file cancers-18-02670-s001.zip › cancers-4422848-supplementary.pdf]

Supplement Figure S1. Progression-Free Survival Model with Subgroups Stratified by Surgical Era (Overlap weighted).

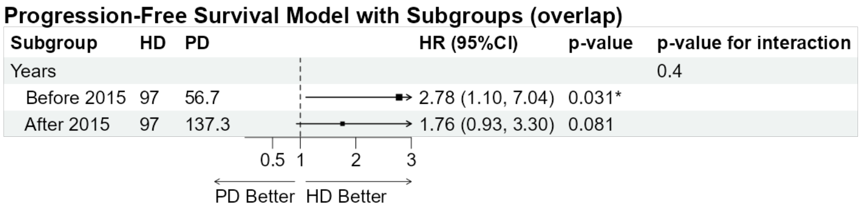

This forest plot presents a subgroup analysis of progression-free survival (PFS) stratified by the year of surgery (before 2015 vs. 2015 and after) following overlap weighting. The adverse prognostic effect of PD on PFS was notably more pronounced in the earlier surgical era (Before 2015: HR = 2.78, P = 0.031) compared to the contemporary era (After 2015: HR = 1.76, P = 0.081), addressing potential confounding from changes in medical practice over the long study period.

Supplementary Figure S2. Cancer-Specific Survival Model with Subgroups Stratified by Surgical Era (Overlap weighted).

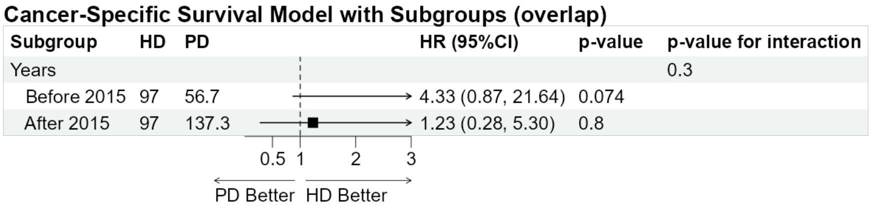

This forest plot presents a subgroup analysis of cancer-specific survival (CSS) stratified by the year of surgery (before 2015 vs. 2015 and after) following propensity score overlap weighting. Consistent with the PFS findings, the elevated cancer-specific mortality risk associated with PD was more pronounced in the earlier surgical era (Before 2015: HR = 4.33, P = 0.074), whereas this survival disadvantage was substantially attenuated in the contemporary era (After 2015: HR = 1.23, P = 0.800).

Supplementary Figure S3. Overall Survival Model with Subgroups Stratified by Surgical Era (Overlap weighted).

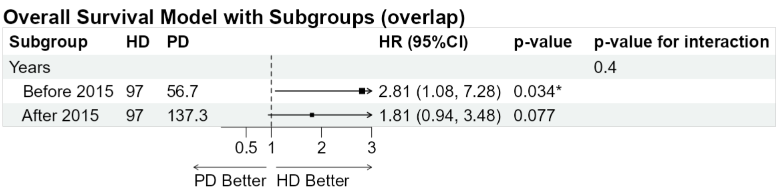

This forest plot presents a subgroup analysis of overall survival (OS) stratified by the year of surgery (before 2015 vs. 2015 and after) following propensity score overlap weighting. Consistent with the PFS

and CSS findings, the elevated overall mortality risk associated with PD was significant in the earlier surgical era (Before 2015: HR = 2.81, P = 0.034), whereas this survival disadvantage was attenuated in the contemporary era (After 2015: HR = 1.81, P = 0.077).

Supplementary Figure S4. Propensity score distributions before and after overlap weighting.

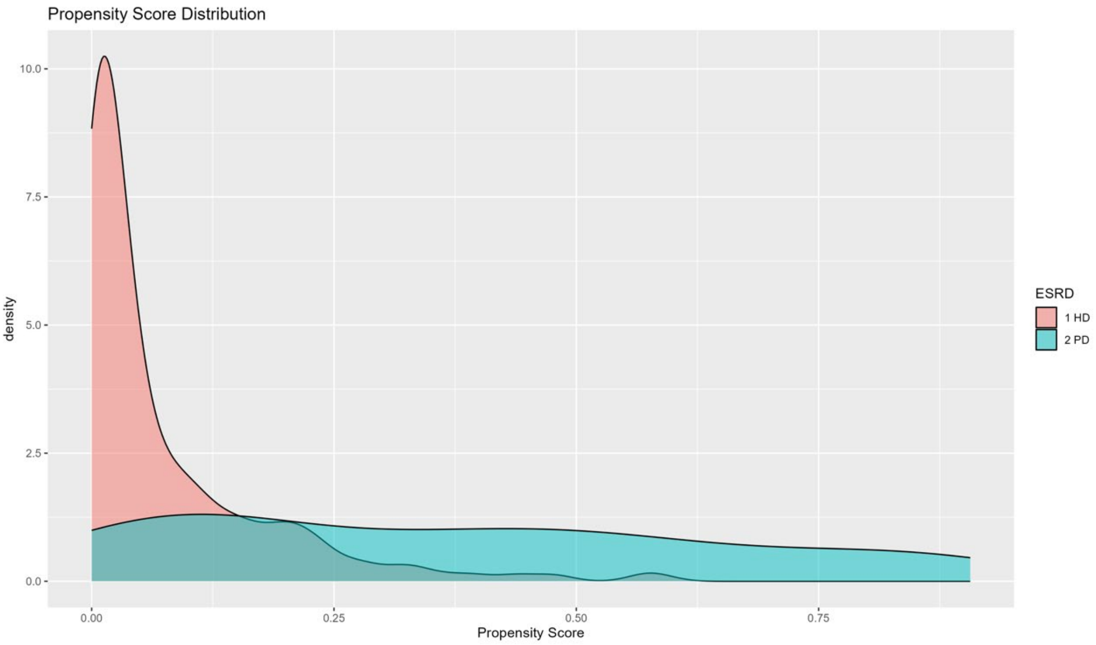

Propensity score distributions before and after overlap weighting. The density plot illustrates the distribution of estimated propensity scores for the HD (red) and PD (blue) groups. Substantial separation is observed between the two groups in the unweighted population, whereas overlap weighting concentrates the analytic weight within the region of common support, where the propensity score distributions of both groups meaningfully overlap.

Supplement Table S1. Univariate Model Cox Regression for Survival

Variables	Levels	OS		CSS		nCS (non-Cancer)		PFS	
		HR	P	HR	P	HR	P	HR	P
ESRD	1 HD	1		1		1		1	
	2 PD	2.15	**0.012***	1.54	0.465	2.47	**0.010***	2.03	**0.019***

		(1.18, 3.91)		(0.48, 4.91)		(1.24, 4.92)		(1.12, 3.67)	
ECOG	0~1	1		1		1		1	
	2~4	2.61 (0.96, 7.11)	0.061	0.69 (0.09, 5.51)	0.723	3.29 (1.13, 9.56)	0.029*	2.25 (0.76, 6.61)	0.141
Sex	1 Male	1		1		1		1	
	2 Female	1.21 (0.55, 2.66)	0.641	1.45 (0.34, 6.09)	0.613	1.13 (0.44, 2.87)	0.804	1.23 (0.55, 2.75)	0.611
Age	Mean (SD)	1.06 (1.03, 1.10)	<0.001*	1.03 (0.96, 1.11)	0.409	1.08 (1.04, 1.12)	<0.001*	1.06 (1.03, 1.10)	<0.001*
HTN	0 No	1		1		1		1	
	1 Yes	0.80 (0.43, 1.50)	0.491	0.47 (0.14, 1.52)	0.207	1.00 (0.46, 2.17)	1.000	0.85 (0.46, 1.59)	0.617
UGI bleeding ulcer	0 No	1		1		1		1	
	1 Yes	0.57 (0.12, 2.77)	0.488	0.19 (0.02, 1.75)	0.142	0.74 (0.14, 3.98)	0.730	0.49 (0.10, 2.34)	0.371
DM	0 No	1		1		1		1	
	1 Yes	1.21 (0.54, 2.70)	0.642	1.46 (0.36, 5.94)	0.599	1.11 (0.41, 2.98)	0.834	1.38 (0.62, 3.08)	0.428
Malignancy	0 No	1		1		1		1	

	1 Yes	0.78 (0.28, 2.18)	0.639	0.80 (0.11, 5.94)	0.824	0.78 (0.25, 2.44)	0.665	0.81 (0.29, 2.27)	0.687
Side	1 Left	1		1		1		1	
	2 Right	0.77 (0.41, 1.46)	0.421	2.13 (0.65, 7.05)	0.215	0.49 (0.22, 1.07)	0.073	0.85 (0.46, 1.59)	0.621
	3 Both	0.12 (0.01, 1.02)	0.052	-		0.14 (0.02, 1.30)	0.084	0.13 (0.02, 1.06)	0.057
Location	1 Renal pelvis	1		1		1		1	
	2 Ureter	0.84 (0.31, 2.30)	0.732	0.20 (0.04, 1.10)	0.065	1.25 (0.38, 4.10)	0.717	0.76 (0.29, 2.00)	0.574
	4 Multiple locations	1.56 (0.70, 3.50)	0.279	1.28 (0.32, 5.14)	0.729	1.76 (0.62, 4.98)	0.286	1.39 (0.64, 2.98)	0.405
Multifocal	0 No	1		1		1		1	
	1 Yes	1.81 (0.90, 3.64)	0.097	1.97 (0.52, 7.44)	0.320	1.75 (0.76, 4.04)	0.188	1.73 (0.88, 3.37)	0.109
Size	1 <2cm	1		1		1		1	
	2 >=2cm	1.08 (0.54, 2.14)	0.834	1.52 (0.40, 5.75)	0.537	0.95 (0.42, 2.11)	0.893	1.14 (0.58, 2.22)	0.708
pT	0 pTis/Ta/T0	1		1		1		1	

	1 pT1	1.83 (0.62, 5.35)	0.271	0.51 (0.04, 5.84)	0.590	2.55 (0.79, 8.27)	0.119	1.75 (0.59, 5.21)	0.315
	2 pT2	1.33 (0.44, 4.04)	0.609	1.03 (0.13, 8.25)	0.981	1.51 (0.43, 5.25)	0.518	1.34 (0.43, 4.13)	0.611
	3 pT3	2.50 (0.86, 7.25)	0.092	3.31 (0.45, 24.22)	0.238	2.03 (0.56, 7.38)	0.284	2.29 (0.77, 6.82)	0.138
pN	0 pNx	1		1		1		1	
	1 pN0	0.54 (0.23, 1.26)	0.155	0.35 (0.10, 1.26)	0.109	0.62 (0.23, 1.66)	0.339	0.54 (0.23, 1.26)	0.153
Grade	1 Low grade	1		1		1		1	
	2 High grade	0.77 (0.31, 1.89)	0.562	39.42 (47.14, 73)	**<0.001***	0.52 (0.21, 1.30)	0.159	0.70 (0.30, 1.62)	0.405
	3 Others	0.18 (0.02, 1.56)	0.121	-		0.18 (0.02, 1.55)	0.119	0.16 (0.02, 1.36)	0.093
Pre-operation Cr level	Mean (SD)	0.96 (0.87, 1.06)	0.439	1.14 (0.91, 1.41)	0.249	0.90 (0.81, 1.00)	0.051	0.98 (0.89, 1.07)	0.582
RNU Method	1 Open	1		1		1		1	
	2 LPS hand-assisted	3.65 (1.3, 10.36)	**0.015***	5.79 (1.47, 22.72)	**0.012***	3.08 (0.9, 10.85)	0.079	3.43 (1.29, 9.08)	**0.013***

	3 Robot assisted	1.12 (0.17, 7.50)	0.910	-		1.40 (0.19, 10.2)	0.742	1.27 (0.25, 6.42)	0.768
	4 Laparoscopy	1.63 (0.55, 4.83)	0.377	2.09 (0.45, 9.60)	0.344	1.52 (0.42, 5.49)	0.527	1.44 (0.52, 4.01)	0.484
Smoking	0 No	1		1		1		1	
	1 Yes	3.20 (1.70, 6.03)	**<0.001***	3.94 (0.75, 20.83)	0.106	1.63 (0.55, 4.86)	0.379	3.70 (1.85, 7.41)	**<0.001***
Previous Nephroureterectomy UC	0 No	1		1		1		1	
	1 Yes	1.23 (0.38, 4.00)	0.731	3.81 (0.95, 15.31)	0.060	0.20 (0.06, 0.61)	**0.005***	1.39 (0.53, 3.63)	0.503
Previous bladder UC	0 No	1		1		1		1	
	1 Yes	2.40 (1.15, 4.98)	**0.019***	1.55 (0.35, 6.84)	0.561	2.72 (1.18, 6.23)	**0.018***	2.51 (1.21, 5.20)	**0.014***
CIS	0 No	1		1		1		1	
	1 Yes	1.53 (0.82, 2.86)	0.181	2.26 (0.69, 7.39)	0.177	1.32 (0.62, 2.82)	0.476	1.50 (0.82, 2.73)	0.185
Pre-operation Urine Cytology	0 Negative	1		1		1		1	

	1 Atypia	1.01 (0.35, 2.89)	0.985	1.14 (0.18, 6.98)	0.891	0.96 (0.30, 3.07)	0.940	1.20 (0.45, 3.18)	0.715
	2 Positive	0.98 (0.33, 2.93)	0.972	2.50 (0.45, 13.97)	0.295	0.74 (0.21, 2.58)	0.640	0.95 (0.34, 2.67)	0.919
	4 No cytology	1.06 (0.40, 2.81)	0.915	3.30 (0.69, 15.77)	0.134	0.72 (0.23, 2.22)	0.564	1.08 (0.43, 2.70)	0.862
LVI	0 No	1		1		1		1	
	1 Yes	1.38 (0.62, 3.05)	0.427	2.63 (0.95, 7.27)	0.062	0.96 (0.24, 3.77)	0.948	1.20 (0.54, 2.67)	0.655
Pre-operative Hydronephrosis	0 No	1		1		1		1	
	1 Yes	1.38 (0.71, 2.68)	0.345	2.13 (0.57, 7.98)	0.261	1.17 (0.54, 2.56)	0.686	1.21 (0.63, 2.31)	0.567
Tumor Necrosis	0 No	1		1		1		1	
	1 Yes	1.50 (0.64, 3.50)	0.346	3.27 (0.81, 13.15)	0.095	0.96 (0.31, 2.91)	0.937	1.43 (0.62, 3.33)	0.404
Chemotherapy for UTUC	0 No	1		1		1		1	
	1 Yes	1.26 (0.54, 2.90)	0.594	2.01 (0.61, 6.68)	0.254	0.99 (0.29, 3.35)	0.982	0.96 (0.40, 2.31)	0.926

Chemotherapy Type for UTUC	0 No	1		1		1		1	
	2 Adjuvant	1.26 (0.54, 2.90)	0.594	2.01 (0.61, 6.68)	0.254	0.99 (0.29, 3.35)	0.982	0.96 (0.40, 2.31)	0.926
Chemotherapy Prescription for UTUC	0 No	1		1		1		1	
	1 Gemcitabine and cisplatin	0.10 (0.02, 0.67)	**0.017***	0.14 (0.01, 1.54)	0.108	0.09 (0.01, 0.91)	**0.041***	0.08 (0.01, 0.55)	**0.010***
	4 Carboplatin-based	1.62 (0.40, 6.54)	0.500	0.00 (0.00, 0.00)	**<0.001***	2.09 (0.52, 8.42)	0.298	1.20 (0.27, 5.27)	0.810
	5 Others	1.74 (0.80, 3.77)	0.162	5.00 (1.71, 14.62)	**0.003***	0.50 (0.06, 3.87)	0.507	1.36 (0.56, 3.33)	0.502
Post-operation Intravesical C/T Instillation	0 No	1		1		1		1	
	1 Intravesical therapy	0.81 (0.26, 2.50)	0.720	1.01 (0.22, 4.57)	0.990	0.73 (0.13, 4.10)	0.720	1.22 (0.49, 3.02)	0.663
Year	Before 2015	1		1		1		1	
	After 2015	1.52 (0.74, 3.13)	0.259	1.35 (0.30, 6.08)	0.698	1.58 (0.69, 3.64)	0.281	2.19 (1.02, 4.70)	**0.045***

** Indicates statistical significance (P < 0.05)*

This table presents the crude hazard ratios (HRs) and 95% confidence intervals (CIs) estimated from univariate Cox proportional hazards regression models for each baseline clinical, pathological, and perioperative covariate in the unweighted study population (N=350). Separate models were constructed to evaluate the unadjusted association of each variable with overall survival (OS), cancer-specific survival (CSS), non-cancer survival (nCS/non-UTUC mortality), and conventional progression-free survival (PFS). . Statistical significance is indicated as P < 0.05

CI: confidence interval; CIS: concomitant carcinoma in situ; Cr: creatinine; CSS: cancer-specific survival; DM: diabetes mellitus; ECOG: Eastern Cooperative Oncology Group performance status; ESRD: end-stage renal disease; HD: hemodialysis; HR: hazard ratio; HTN: hypertension; LPS: laparoscopic; LVI: lymphovascular invasion; nCS: non-cancer survival (non-UTUC mortality) ; NxUx / RNU: radical nephroureterectomy; OS: overall survival; PD: peritoneal dialysis; PFS: progression-free survival; SD: standard deviation; UC: urothelial carcinoma; UGI: upper gastrointestinal

Supplement Table S2. Univariate Fine-Gray Hazard Model for Survival

Variables	Levels	CSS		nCS (non-Cancer)		PFS	
		HR	P	HR	P	HR	P
ESRD	1 HD	1		1		1	
	2 PD	1.86 (0.70, 4.91)	0.211	1.38 (0.79, 2.43)	0.258	1.67 (1.03, 2.72)	**0.038***
ECOG	0~1	1		1		1	
	2~4	1.04 (0.31, 3.46)	0.947	2.34 (1.42, 3.84)	**0.001***	2.33 (1.49, 3.64)	**<0.001***
Sex	1 Male	1		1		1	
	2 Female	0.60 (0.28, 1.29)	0.191	0.90 (0.61, 1.34)	0.600	0.75 (0.53, 1.06)	0.103

Age	Mean (SD)	1.00 (0.96, 1.04)	0.907	1.07 (1.04, 1.09)	**<0.001***	1.07 (1.05, 1.09)	**<0.001***
HTN	0 No	1		1		1	
	1 Yes	1.44 (0.66, 3.13)	0.365	1.46 (0.99, 2.14)	0.057	1.59 (1.13, 2.25)	**0.008***
UGI bleeding ulcer	0 No	1		1		1	
	1 Yes	0.33 (0.04, 2.41)	0.273	1.13 (0.61, 2.11)	0.696	0.83 (0.46, 1.50)	0.532
DM	0 No	1		1		1	
	1 Yes	1.89 (0.80, 4.47)	0.148	1.24 (0.76, 2.04)	0.390	1.71 (1.13, 2.60)	**0.012***
Malignancy	0 No	1		1		1	
	1 Yes	0.51 (0.12, 2.15)	0.360	1.77 (1.08, 2.91)	**0.025***	1.49 (0.93, 2.38)	0.095
Previous Nephroureterectomy UC	0 No	1		1		1	
	1 Yes	1.54 (0.65, 3.65)	0.325	0.72 (0.43, 1.23)	0.233	0.96 (0.62, 1.50)	0.874
Previous bladder UC	0 No	1		1		1	

	1 Yes	1.10 (0.46, 2.59)	0.833	1.13 (0.73, 1.75)	0.571	1.35 (0.92, 1.97)	0.127
RNU Method	1 Open	1		1		1	
	2 LPS hand-assisted	2.82 (1.09, 7.28)	**0.032***	0.89 (0.54, 1.47)	0.651	1.31 (0.86, 2.00)	0.214
	3 Robot assisted	0.66 (0.08, 5.36)	0.697	0.61 (0.24, 1.54)	0.294	0.89 (0.42, 1.87)	0.751
	4 Laparoscopy	1.30 (0.47, 3.58)	0.616	0.89 (0.57, 1.38)	0.593	0.96 (0.64, 1.44)	0.842
Year	Before 2015	1		1		1	
	After 2015	1.21 (0.56, 2.64)	0.624	1.01 (0.69, 1.49)	0.956	1.73 (1.19, 2.50)	**0.004***

** Indicates statistical significance (P < 0.05)*

This table summarizes the crude subdistribution hazard ratios (sHRs) and 95% confidence intervals (CIs) derived from univariate Fine-Gray subdistribution hazard regression models in the unweighted study population (N=350)

. These models were specifically performed to account for the confounding influence of competing events in this dialysis population

CI: confidence interval; CSS: cancer-specific survivl; ECOG: Eastern Cooperative Oncology Group performance status; ESRD: end-stage renal disease; HD: hemodialysis; HTN: hypertension; nCS: non-cancer survival (non-UTUC mortality) ; PD: peritoneal dialysis; RNU: radical nephroureterectomy;

sHR: subdistribution hazard ratio (presented as HR in the table) ; UC: urothelial carcinoma; UGI: upper gastrointestinal
